# Pathogen priming alters host transmission potential and predictors of transmissibility in a wild songbird species

**DOI:** 10.1128/msphere.00886-24

**Published:** 2025-03-10

**Authors:** A. E. Leon, A. E. Fleming-Davies, J. S. Adelman, D. M. Hawley

**Affiliations:** 1 Department of Biological Sciences, Virginia Tech, Blacksburg, Virginia, USA; 2 Department of Biology, University of San Diego, San Diego, California, USA; 3 Department of Biological Sciences, University of Memphis, Memphis, Tennessee, USA; University of Kentucky College of Medicine, Lexington, Kentucky, USA

**Keywords:** acquired protection, reinfection, transmission, *Mycoplasma gallisepticum*, virulence evolution

## Abstract

Pathogen reinfections occur widely, but the extent to which reinfected hosts contribute to ongoing transmission is often unknown despite its implications for host-pathogen dynamics. House finches (*Haemorhous mexicanus*) acquire partial protection from initial exposure to the bacterial pathogen *Mycoplasma gallisepticum* (MG), with hosts readily reinfected with homologous or heterologous strains on short timescales. However, the extent to which reinfected hosts contribute to MG transmission has not been tested. We used three pathogen priming treatments—none, intermediate (repeated low-dose priming), or high (single high-dose priming)—to test how prior pathogen priming alters the likelihood of transmission to a cagemate during index bird reinfection with a homologous or heterologous MG strain. Relative to unprimed control hosts, the highest priming level strongly reduced maximum pathogen loads and transmission success of index birds during reinfections. Reinfections with the heterologous strain, previously shown to be more virulent and transmissible than the homologous strain used, resulted in higher pathogen loads within high-primed index birds and showed higher overall transmission success regardless of host priming treatment. This suggests that inherent differences in strain transmissibility are maintained in primed hosts, leading to the potential for ongoing transmission during reinfections. Finally, among individuals, transmission was most likely from hosts harboring higher within-host pathogen loads. However, associations between disease severity and transmission probability were dependent on a given bird’s priming treatment. Overall, our results indicate that reinfections can result in ongoing transmission, particularly where reinfections result from a highly transmissible strain, with potential implications for virulence evolution.

**IMPORTANCE:**

As COVID-19 dramatically illustrated, humans and other animals can become infected with the same pathogen multiple times. Because individuals already have defenses against pathogens that their immune systems encountered before, reinfections are likely less contagious to others, but this is rarely directly tested. We used a songbird species and two strains of its common bacterial pathogen to study how contagious hosts are when their immune systems have some degree of prior experience with a pathogen. We found that reinfected hosts are not as contagious as initially infected ones. However, the more transmissible of the two strains, which also causes more harm to its hosts, was able to multiply more readily than the other strain within reinfected hosts and was more contagious in both reinfected and first-infected hosts. This suggests that reinfections might favor more harmful pathogen strains that are better able to overcome immune defenses.

## INTRODUCTION

Reinfections are a common feature of many host-pathogen systems (1–4), including those of humans (e.g., SARS-CoV-2 [5]). The apparent pervasiveness of reinfections is somewhat surprising given that vertebrate immune systems harbor specific immune memory (6), allowing hosts to respond more rapidly and effectively to reinfection with the same pathogen. Nonetheless, the immune protection generated by prior pathogen infection is often incomplete and/or wanes over time (7, 8), such that some degree of host reinfection is possible (9–11). While the ubiquity of reinfection in many vertebrate host-pathogen systems is now well appreciated (11), few studies directly quantify the transmission potential of reinfected hosts and whether it varies between pathogen strains or with the degree of host prior exposure. Such questions are key for characterizing epidemiological dynamics that account for variability in infection-derived immunity (reviewed in references 11, 12) and for understanding the selective pressures on pathogens infecting hosts with acquired but incomplete immunity (10, 13, 14).

Because initial pathogen infection generates acquired immunity with some degree of specificity for many vertebrate hosts, reinfections with the same pathogen generally result in lower pathogen loads, reduced disease severity, and/or increased survival relative to hosts infected for the first time, which have no acquired protection (e.g., reference 15). In turn, lower pathogen loads during reinfection are predicted to reduce a reinfected host’s transmission potential relative to a host infected for the first time. In two studies that experimentally infected mice with *Plasmodium chabaudi* parasites and then rapidly treated them to create “immunized” mice, a three- to fourfold reduction in the density of transmission-stage parasites was documented in previously immunized versus non-immunized mice (14, 16). Interestingly, reductions in within-host burdens of transmission-stage parasites due to prior immunization were equivalent for homologous versus heterologous challenge strains of *P. chabaudi*, though heterologous strains were better able to successfully transmit to mosquito vectors (16). This discrepancy underscores the need to measure transmission success *per se* from reinfected hosts, rather than relying solely on variation in pathogen or parasite burdens as a metric of infectiousness.

Numerous studies of vaccinated hosts across diverse taxa also find that hosts challenged with the specific pathogen they were vaccinated against show lower transmission ability relative to unvaccinated hosts (13, 17–19). Notably though, the effects of vaccination on host transmission probability can also vary across pathogen strains, in some cases, in association with strain virulence (defined here as the average reduction in host fitness due to infection with a given strain). For example, vaccination of chickens for Marek’s virus reduced their transmission potential (relative to unvaccinated hosts) for a low-virulence strain of virus but actually enhanced the transmission potential of high-virulence strains (13). This occurred because vaccination against Marek’s virus generates incomplete immunity, protecting hosts from virus-induced mortality but not viral replication; together, this extends the infectious periods for virulent strains in vaccinated versus unvaccinated hosts, the latter of which rapidly succumb to virulent strains, often prior to transmitting (13). Thus, in addition to transmission success, it is important to understand how immunization from prior infection or vaccination influences disease severity, which may determine reinfected host survival.

Other characteristics of pathogen strains, in addition to virulence, can potentially influence transmission potential during reinfection. Across taxa, inherent differences between strains in within-host replication rates are often positively associated with transmission rates (20), suggesting that strain characteristics that influence within-host replication can, at least in some cases, predict transmission potential. High within-host replication rates are, in turn, positively associated with strain virulence in many systems (20, 21); for example, across 10 parasite clones of *P. chabaudi* infecting laboratory mice, parasite clone growth, virulence, and transmissibility were positively related (14, 16). Although strains infecting immunized mice showed overall reductions in all three pathogen fitness traits, the positive relationships between clonal growth, virulence, and transmissibility persisted in immunized hosts, potentially favoring virulent strains able to generate sufficient within-host growth and, thus, transmissibility in immunized hosts (22, 23). In addition to traits such as virulence and transmissibility, antigenic relationships among strains can determine the ability to sufficiently overcome host immune protection generated by initial infection: for example, serum from humans previously infected with a variant of SARS-CoV-2 showed stronger neutralization ability against homologous versus heterologous viral variants (24). Overall, such studies suggest that transmission success during reinfection can be strain specific, with transmission more likely during reinfections with heterologous and/or more virulent strains (16, 25, 26), provided such strains can better escape or overcome the acquired protection present in immunized hosts (22).

In addition to strain characteristics, the extent to which reinfected hosts transmit is likely dependent on the strength of acquired protection harbored by an individual at the time of reinfection. For example, the degree of SARS-CoV-2 infectiousness (measured as attack rate in close contacts) during reinfections or breakthrough infections was lowest for individuals who had been vaccinated and also experienced prior natural infection (27). Furthermore, serum collected from individuals with prior malaria infections in quick succession showed the strongest transmission-blocking immunity against *Plasmodium vivax* (28). Natural host-pathogen systems are inherently variable in the extent of initial pathogen exposure that hosts experience (29, 30). Given that the strength of protection acquired from initial infection can vary with the dose (28, 29, 31) and frequency (28, 32) of prior pathogen exposure that a host experiences, variation in the extent of pathogen priming is predicted to influence the likelihood of ongoing transmission during reinfection.

Wildlife systems in which reinfections are common, such as the bacterial pathogen *Mycoplasma gallisepticum* (hereafter, “MG”) of house finches (*Haemorhous mexicanus*), allow experimental tests of how the extent of pathogen priming alters host transmission potential during reinfection with distinct pathogen strains (here, homologous versus heterologous). MG causes seasonal epidemics of mycoplasmal conjunctivitis in house finch populations across most of North America (33). MG is transmitted both directly and via finch contact with contagious surfaces at bird feeders (34). Because this obligate pathogen is short-lived outside of the host (35), finches experience variable levels of exposure at contaminated feeders. Diseased finches in the wild recover at high rates (36), and experimental studies show that reinfections are characterized by significantly lower pathogen loads and disease severity relative to first infections (37). Nonetheless, recovered individuals remain susceptible to reinfection with both homologous and heterologous strains (10, 37, 38), with little evidence for additional protection associated with reinfection strain homology (10).

While prior work suggests that reinfections are common in this host-pathogen system, the likelihood and severity of reinfection vary with both the degree of initial pathogen priming and the identity of the reinfecting strain. Leon and Hawley (39) experimentally varied the degree of pathogen priming experienced by finches, finding that a single high-dose MG priming treatment results in stronger host protection from homologous reinfection than intermediate degrees of MG priming such as repeated, low-dose exposures. A follow-up study (40) using similar priming treatments followed by challenge with one of three distinct MG strains found that reinfections of primed hosts by a heterologous, more virulent MG strain were associated with higher within-host pathogen loads relative to MG strains with lower virulence and within-host replication rates (10, 40). Because within-host pathogen loads serve as a potential proxy for transmission likelihood, such results suggest that reinfection with a heterologous, more virulent strain may result in higher transmission in this system, but work to date has not directly assessed transmission success. Given that prior studies have found discrepancies between the effects of host immunization on proxies of transmission (such as the density of transmission-stage parasites) versus direct measures of transmission success (16), it is key to examine the effects of pathogen priming on between-host transmission success to fully uncover the importance of reinfections for pathogen ecology and evolution.

While no studies have examined MG transmission potential during host reinfection, several past studies quantified transmission in immunologically naive house finches. Among MG strains, higher within-host pathogen loads are associated with higher transmission rates (41), and within a given strain, higher pathogen loads result in greater deposition of MG onto bird feeder surfaces (42). Host disease severity is also associated with transmission potential (43), with birds with more severely inflamed conjunctiva more likely to transmit to cagemates, even when accounting for their higher pathogen loads (43–45). Given that reinfections in this system (10, 39) and others (14, 15) are characterized by significant reductions in disease severity relative to hosts infected for the first time, the respective roles of pathogen load and disease severity in predicting transmission potential for pathogen-naive and reinfected hosts are key for understanding the selective pressures on pathogens to cause higher disease severity (i.e., virulence) in hosts.

Here, we test how pathogen priming alters transmission potential for hosts reinfected with one of two strains (homologous versus heterologous) and quantify individual correlates of transmission success (disease severity and pathogen load). Although we did not have sufficient strain replication to isolate the effects of strain virulence *per se* on transmission success, our reinfections used two strains that differ in virulence (10, 46), within-host replication rate (40, 46), and transmission potential (41). We specifically selected a more virulent strain as our heterologous strain for reinfections because MG strains collected from wild house finches have increased in virulence over time (46, 47), with more virulent strains associated with higher transmissibility (41, 44). Thus, our experimental design mimicked a natural population in which individuals are most likely to be reinfected by either an endemic strain homologous to that which the host recently recovered from or by an invading heterologous strain that has higher inherent transmissibility and virulence (44). To create variation in the degree of priming experienced by hosts, we varied the number and concentration of priming doses with a single MG strain (VA94) to create three priming levels: none, intermediate, or high. After recovery, we (re)-inoculated hosts and assessed the effects of priming treatment on transmission success to a naive cagemate during reinfection with one of two MG strains (the homologous strain, VA94, or the heterologous strain, NC06). Based on prior work (39, 40), we predicted the highest priming level would result in the lowest host transmission potential. We also predicted that, consistent with prior work (40, 41), the heterologous, more virulent strain (NC06) would have higher overall transmission success, regardless of host priming treatment, with potential interactive effects of host priming and strain identity on transmission, as detected in a rodent-malaria system (16). Finally, based on prior work (44, 45), we predicted that disease severity would correlate with transmission potential in naive hosts, but less so for reinfected hosts, which typically show stronger protection from disease versus pathogen loads (39).

## MATERIALS AND METHODS

### Experimental design

**Fig 1:**
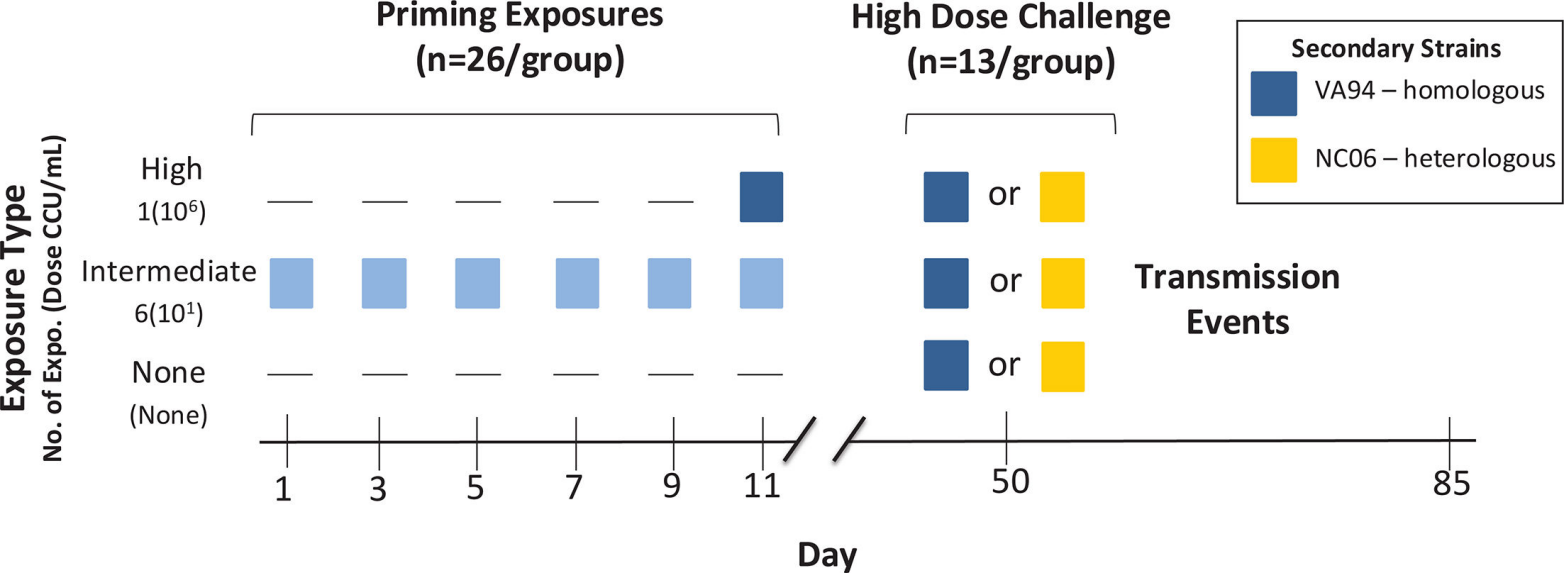
Experimental design and timeline. Index individuals (housed alone for the priming portion) were given one of three priming treatments (*y*-axis) with the *M. gallisepticum* strain VA94 (blue squares), with treatments varying by the number of priming exposures and dose (the color gradient indicates variation in dose, with more intense color indicating increasing dose concentration). On day 39 post-priming treatment, all index birds were challenged with one of two strains: one homologous to that used in priming exposures (VA94, shown in blue) or a heterologous strain (NC06, shown in yellow) and then pair-housed with an MG-naive cagemate to assess pairwise transmission success.

Seventy-eight captive, MG-naive house finches (see supplemental material) were randomly assigned to one of three priming treatments (*n* = 26/group) that varied in both dose of pathogen exposure and total number of exposures (Fig. 1). These priming levels—a negative control group (no priming), a low-dose repeat exposure group (“intermediate” priming), and a single high-dose exposure group (“high” priming)—were selected because they produced the greatest range in acquired protection in prior work, as measured by pathogen loads and disease severity during reinfection challenge (40). Inoculations for the intermediate priming group, which received six sequential exposures at 10^1^ color-changing units (CCU)/mL, were given every other day and designed so that all groups received their final inoculation on the same day to allow for the same window of recovery prior to challenge (Fig. 1).

Individuals were housed alone during priming treatment and given 39 days to recover from priming exposures, consistent with prior work (40). All birds then received a secondary, high-dose challenge with either a homologous or heterologous strain of MG (*n* = 13 pairs/group; Fig. 1). At the time of the secondary challenge, each of these 78 individuals was pair-housed with an MG-naive cagemate to determine how pairwise transmission success varies with both the degree of pathogen priming and strain identity. Hereafter, individuals who acted as “transmitters” are referred to as “index” birds, and their immunologically naive cagemates used to assess transmission potential are referred to as “naive” birds. All transmission pairs were the same sex to avoid any potential contributions of mating behaviors to transmission dynamics. Sex ratios of index birds were even for priming treatment groups (13:13, male:female). For secondary treatments, all transmission pairs were same sex, but because groups had a sample size of 13 (Fig. 1), sex ratios were randomly assigned as either 6:7 male:female pairs or 7:6 female:male pairs. See supplemental material for capture and housing details.

### Pathogen inoculations

Two strains (with strain defined here as a unique genetic variant [48]) were selected based on previous work demonstrating differences in the maximum and average pathogen loads and virulence they produce in immunologically naive house finches (10, 46). The house finch MG strain “VA94” (7994-1 7P 12/12/09) (49) was used for all priming exposures, whether intermediate or high priming (Fig. 1). Secondary challenge inoculations were all at high dose and varied only in strain identity: either the priming strain (homologous) or a heterologous strain “NC06” [2006.080-5 (4P) 7/26/12] used to represent a hypothetical invading strain with higher transmissibility. Inoculations were administered via droplet installation directly into the conjunctiva (70 µL total volume across both conjunctivas) via micropipette (see supplemental material). The negative control (sham inoculation) group received 70 µL of sterile media.

### Disease severity and pathogen load

Disease severity was assessed by scoring the degree of visible inflammation, eversion, and exudate in conjunctival tissue (50) on a scale of 0–3 for each eye and summing across eyes per individual within a given sampling date. Scoring was done blind to treatment. Pathogen load was assessed via swabbing of conjunctival tissue and MG-specific qPCR (see supplemental material), with loads log10 transformed for analysis.

Index birds were eye scored and sampled for pathogen load on post-secondary inoculation days (PSID) 4, 7, 14, 21, and 28. To obtain high-resolution data on transmission timing, naive cagemates were eye scored daily on PSID 5 through 18 and then on days 21, 23, 25, 28, 32, and 35. Additionally, to ensure all individuals were still naive to MG just prior to the start of the experiment, we sampled for eye score and pathogen load on pre-inoculation day 19, as well as pre-challenge day 4 to obtain baseline data prior to re-inoculation. Responses to priming exposure levels have been previously examined (39) and are not included here.

### Transmission

Pairwise transmission was quantified as successful when a previously naive cagemate developed scorable eye lesions (>0). Although the use of eye score as the assay for transmission can miss low-level, subclinical infections, prior work comparing the two metrics (34) showed that using eye score as the transmission metric robustly captures naive individuals with minimum pathogen loads (>1,349 copies across both conjunctiva) considered to be infectious in this system. Furthermore, the use of eye score eliminates potential false positives, which are known to occur in our qPCR assay (39).

### Analyses

All analyses were done using the statistical software R (51). In models where interactions were not significant, overall level effects were analyzed using Type II Likelihood Ratio tests using the car package in R (52). Whenever significant interactions were present, effects were analyzed using a type III likelihood ratio (52). *Post hoc* pairwise differences for significant interactions of interest were generated using the emmeans function and a Tukey adjustment for multiple comparisons.

#### Within-host responses

Because we were interested in the extent to which transmission success was a function of within-host responses, we analyzed both disease severity and pathogen load during the secondary challenge of index birds with distinct priming treatments. As in our prior work (10), we ensured the independence of repeated-measures data by analyzing only the maximum eye score and pathogen load for each index bird across four post-secondary challenge time points (7, 14, 21, and 28). For both models, fixed effects included priming treatment, secondary strain, and an interaction between the two effects (removed if not significant). To account for the non-continuous nature of score data, the maximum eye score was treated as an ordinal factor and analyzed using cumulative link models (CLMs) in the ordinal package (53). Maximum pathogen load was analyzed using a generalized linear model with a gamma distribution, chosen because pathogen load was positively skewed, and inverse link function (lme4 package in R [54]). Prior to analysis, a minuscule value of 1 × 10^−13^ was added to each maximum load to meet assumptions of the gamma distribution, which only allows positive values.

#### Transmission

Pairwise transmission (Y or N), assessed via any visible eye lesions in cagemates, was analyzed using logistic regression with binomial distribution and a logit link function. Fixed effects included priming treatment and secondary challenge strain. An interaction between priming exposure and secondary strain was tested but not included in the final model, as the interaction was not significant (LR = 4.48, df = 2, and *P* = 0.11) and the model including an interaction was a poor fit to the data.

To determine which host factors (eye score, pathogen load, or both) are predictive of transmission success across priming treatments, pairwise transmission was also analyzed across individuals using a second logistic regression with binomial distribution and a logit link function. Because our analyses of eye score and pathogen load indicated that priming treatment influences each host response somewhat distinctly, we included priming treatment in interaction with maximum pathogen load and eye score in the model. Although previous work found correlations between pathogen load and eye score across MG strains (46), in this study, the two variables were correlated at a level of 0.54 (Pearson correlation coefficient) across individuals, allowing us to include these variables as independent fixed effects in our model (55).

## RESULTS

### Index bird within-host responses

**Fig 2:**
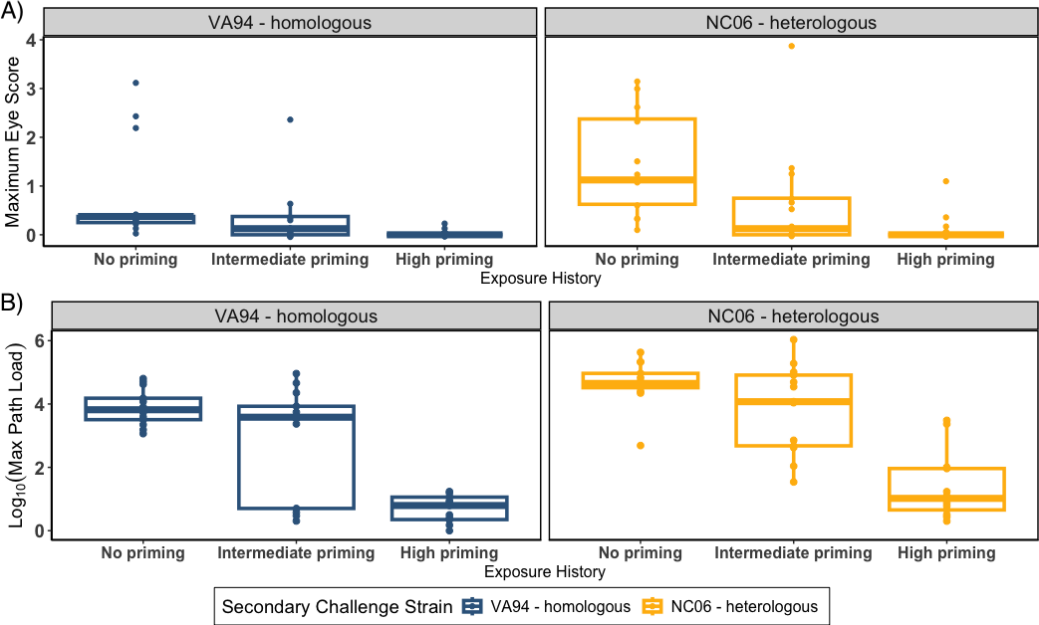
(A) Maximum eye scores and (B) pathogen loads after secondary challenge with one of two *Mycoplasma gallisepticum* strains (left: homologous VA94, blue; right: heterologous NC06, yellow) in index birds with distinct pathogen priming histories (*x*-axis: none, intermediate, or high). Each point represents maximum responses for each individual over four sample points. While eye scores are visualized as continuous here, they were analyzed as ordinal factors to account for their non-continuous distribution.

All birds recovered from priming-induced disease (eye scores = 0) by the time of the reinfection challenge on day 39. The level of pathogen priming that an index bird received significantly predicted host disease severity (i.e., maximum eye scores) during reinfection challenge, with the lowest disease in birds that received high pathogen priming prior to challenge (CLM estimates: intermediate priming: −1.48 ± 0.71; high-dose priming: −3.34 ± 0.93; priming treatment LR Chisq = 31.8, df = 2, and *P* < 0.0001; Fig. 2A). As expected, the more virulent NC06 strain produced higher maximum disease severity in birds than VA94 (strain[NC06]: 1.59 ± 0.72), but secondary strain was not significant in the overall model (secondary strain LR = 2.15, df = 1, and *P* = 0.14). There was no significant interaction between priming treatment and secondary strain identity on disease severity in index birds (priming:secondary LR = 5.10, df = 2, and *P* = 0.078), suggesting that the effects of priming on disease severity were largely similar between the strains.

Pathogen loads just prior to reinfection challenge (day −4) were slightly elevated in index birds that received high priming treatment (Fig. S1), suggesting complete clearance of high-dose priming had not universally occurred by the time of reinfection challenge. Nonetheless, birds that received high priming reached lower maximum pathogen loads during the reinfection challenge than did birds with intermediate or no priming, indicating that residual pathogen from priming treatments was outweighed by the effects of the protection acquired from priming (GLM assuming gamma distribution [parameters on *inverse* scale]: intermediate priming: 0.049 ± 0.036; high priming: 0.616 ± 0.10; and priming treatment LR = 68.42, df = 2, and *P* < 0.0001; Fig. 2B). Priming treatment also interacted with strain identity to predict pathogen loads during reinfection (priming:secondary strain LR = 11.68, df = 2, and *P* = 0.0029). Specifically, in index birds given high priming, hosts challenged with the heterologous, more virulent strain NC06 harbored significantly higher maximum pathogen loads than those challenged with VA94 (Table 1). Secondary strain did not have a significant main effect on pathogen loads (secondary strain LR = 0.245, df = 2, and *P* = 0.62).

**TABLE 1:** Pairwise *post hoc* comparisons for the significant interactive effect of priming (“prim.”) treatment (none, intermediate: “int,” or high) and secondary strain (VA94 or NC06) on maximum pathogen loads following secondary challenge^*a*^

Priming: strain	No priming VA94	Int. prim. VA94	High prim. VA94	No priming NC06	Int. prim. NC06	High prim. NC06
No priming VA94	5.37	0.735	<0.001**	*0.996*	1.000	0.0007**
Intermediate VA94	−0.049	4.24	<0.001**	0.436	*0.656*	0.0218*
High priming VA94	−0.616	−0.567	1.25	<0.001**	<0.0001**	0.0119*
No priming NC06	*0.015*	0.064	0.631	5.83	0.999	0.0002**
Intermediate NC06	0.004	*0.054*	0.620	−0.011	5.49	0.0005**
High priming NC06	−0.234	−0.185	*0.382*	−0.249	−0.239	2.38

^*a*^ Interactive comparisons (strain differences within priming treatment) are in italics for ease of visualization. Cells above the shaded diagonal give Tukey-adjusted *P*-values (*P < 0.05 and **P < 0.01), while cells below the diagonal give estimates for each pairwise comparison. Diagonal cells (shaded) show predicted pathogen loads (emmeans) for each combination on the response scale. Within the high priming treatment only, birds reinfected with NC06 had significantly higher pathogen loads than birds infected with VA94.

### Transmission success

**Fig 3:**
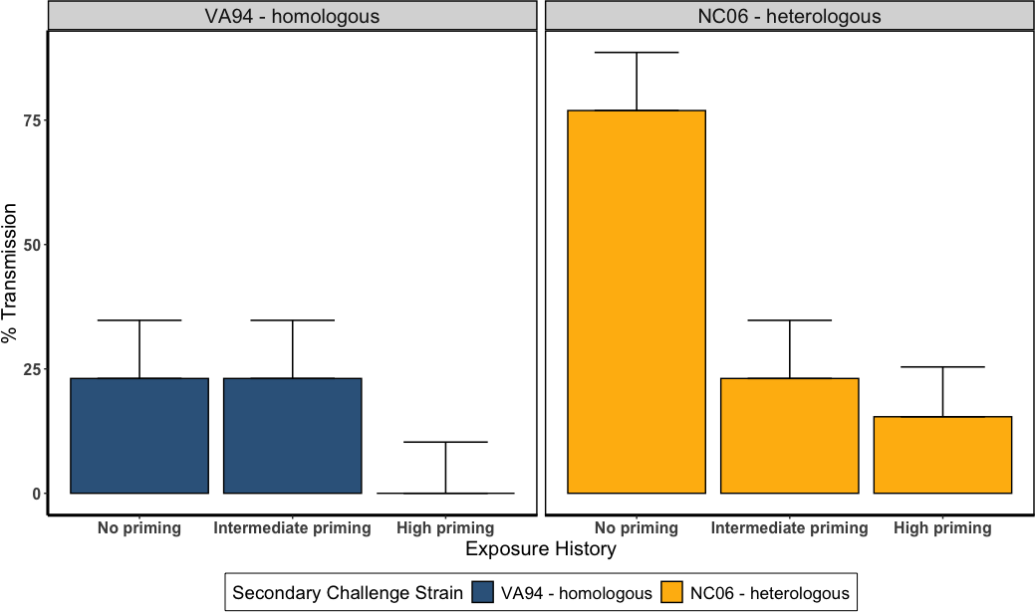
Pairwise transmission of *Mycoplasma gallisepticum* from captive house finches with variable pathogen priming (*x*-axis) that were experimentally inoculated with one of two pathogen strains (left: VA94—homologous, shown in blue; right: NC06—heterologous, shown in yellow). Percentage of transmission (*y*-axis) from index birds (transmitters) was measured in pathogen-naive cagemates. Higher degrees of priming reduced an index bird’s transmission potential to its cagemate for both strains, though the NC06 strain produced higher transmission regardless of host priming. Each treatment group had 13 pairs. Error bars represent binomial standard errors calculated using sample size.

Pairwise transmission success was significantly lower, relative to unprimed birds, in reinfected index birds that received intermediate or high priming prior to secondary challenge, with the strongest reduction in transmission potential in the high priming group (logistic regression with a binomial distribution: intermediate priming: −0.7703 ± 0.381; high priming: −1.581 ± 0.470; LR = 13.63, df = 2, and *P* = 0.001; Fig. 3). The heterologous, higher virulence NC06 strain had higher overall transmission success than the VA94 strain (NC06 strain: 0.8627 ± 0.351; LR = 6.45, df = 1, and *P* = 0.011; Fig. 3). Although NC06 was notably the only strain with detectable transmission from high priming index birds, the low overall transmission success from hosts with high priming (2/13 pairs for NC06, relative to 0/13 for VA94) made any statistical interactions between secondary strain identity and priming treatment challenging to estimate.

### Host factors predictive of transmission

**Fig 4:**
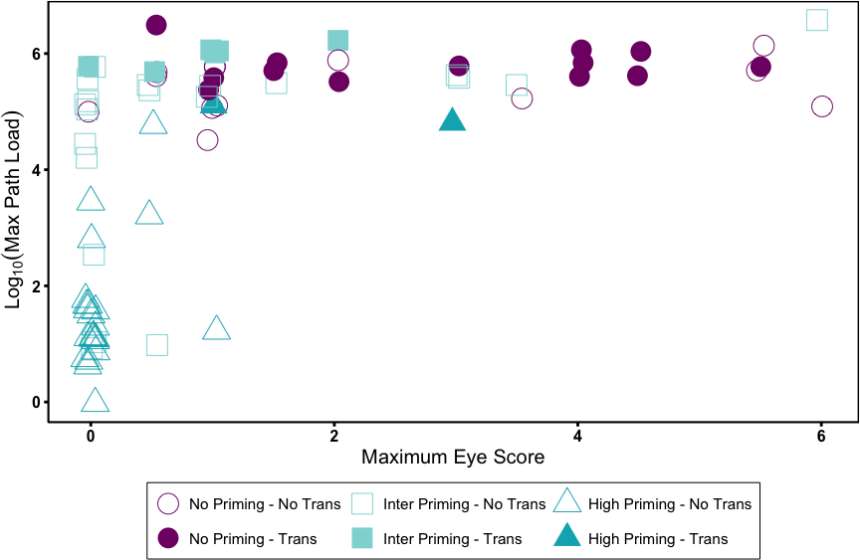
Successful pairwise transmission of *Mycoplasma gallisepticum* (filled symbols) from index house finches (*n* = 78) given various priming treatments (symbols: unprimed birds, circles; intermediate priming, squares; and high priming, triangles) was a function of the pathogen load of the index bird (*y*-axis), with successful transmission occurring only from index birds above a threshold pathogen load of log_10_ ~ 4.5. There were also significant effects of index bird maximum eye score (*x*-axis) in interaction with pathogen priming treatment, with higher eye scores associated with lower transmission success at intermediate priming levels (squares), while for high priming birds (triangles), intermediate eye scores, which were the highest maximum reached for this treatment, were positively associated with transmission likelihood.

The model of index bird transmission (Y/N) with the strongest support included an index bird’s maximum pathogen loads during the secondary challenge, as well as the interaction between the index bird maximum eye score and priming treatment. Specifically, across all priming treatments, index bird pathogen loads were positively associated with transmission probability (logistic regression: pathogen load: 3.93 ± 1.52, LR = 14.58, df = 1, and *P* < 0.001). In contrast, the relationship between an index bird’s maximum eye score and transmission success depended on a bird’s priming treatment (eye score × priming treatment: LR = 6.70, df = 2, and *P* = 0.035; interaction visualized in Fig. 4). For birds given intermediate priming, higher maximum eye scores during reinfection were associated with slightly lower transmission (intermediate priming × score: −0.938 ± 0.601), while a positive effect of eye score was estimated for high-primed birds (high priming × score: 46.1 ± 5,055.7), although the model parameter could not be estimated with any precision due to the low number of high-primed birds with detectable eye score. The three-way interaction between pathogen load, disease severity, and priming treatment was not significant (*P* > 0.99) and was removed from the final model.

## DISCUSSION

We show that pathogen priming significantly reduces transmission success during reinfections relative to birds infected for the first time. Nonetheless, reinfected hosts were still associated with a notable degree of successful pairwise transmission, although the extent of successful transmission varied with both the reinfecting pathogen strain and priming level. Together, our findings suggest that reinfections can meaningfully contribute to transmission dynamics in this system, particularly in cases that approximate our intermediate priming treatment, such as when pathogen priming in a population is variable, or acquired protection from previous infection has waned over time (37). As such, variation in the degree of acquired protection among hosts in natural populations, in systems where reinfections are common, can be an important impediment to transmission, placing selective pressure on invading pathogen strains to overcome host-acquired protection.

Our main objective was to determine how the degree of pathogen priming, and thus the degree of acquired protection, alters host transmission potential during reinfection. For both strains examined, we found that the highest priming level resulted in strong reductions in transmission potential relative to unprimed hosts. These results are consistent with the effects of host immunization on transmission likelihood in other key systems where this question has been examined, including malaria in mouse models (16) and humans (28), and SARS-CoV-2 in humans (27, 56–58). Together, these results demonstrate that prior pathogen infection or vaccination at levels sufficient to generate acquired protection can have strong effects on epidemic dynamics in wild populations, as documented in vaccinated human populations (e.g., reference 59), and likely provide strong selection on pathogens circulating in host populations where many individuals have acquired protection (13).

Our results also suggest that the degree of pathogen priming a host experienced has key effects on transmission success, potentially akin to the additive effects of vaccination and prior natural infection on the infectiousness of breakthrough SARS-CoV-2 infections (27). Here, while our highest priming treatment caused strong reductions in transmission success for both strains, there were apparent, though not statistically significant, differences between the two strains in response to our intermediate priming level. The homologous VA94 strain showed no difference in transmission success between the no priming and intermediate priming levels. In contrast, the intermediate priming treatment resulted in fewer successful transmission events relative to unprimed birds for the heterologous NC06 strain. Future work should incorporate a low priming treatment, such as a single, low priming dose (39), to test whether there are strain-specific effects of lower degrees of pathogen priming on transmission in this system.

We also detected overall strain differences in transmission success akin to prior work (41), with the NC06 strain showing higher transmission success than VA94, regardless of a host’s priming treatment. For within-host pathogen loads, however, the effects of strain were dependent on the host priming background, with the NC06 strain reaching significantly higher within-host pathogen loads than the VA94 strain within the high-priming group. This pathogen load advantage for NC06 may explain why there was some successful transmission (~15%) of the NC06 strain even from hosts with the high priming level, whereas there were no instances of successful transmission of the VA94 strain from hosts with the same high priming level. Overall, our findings align with mouse-malaria studies showing that parasite clone differences in growth rate and transmission are largely maintained in immunized hosts, despite overall reductions in average growth and transmission (14, 16).

The use of only two strains (one homologous and one heterologous, more virulent strain) in this study precludes our ability to directly determine what aspects of strain identity, whether heterology, virulence, or both, influenced transmission potential in our study and requires us to limit our conclusions to the two specific strains examined. While further studies are needed with larger numbers of strains, several lines of evidence suggest that heterology is unlikely to explain the transmission advantage of NC06 during reinfections: first, heterology is only a relevant mechanism in primed hosts, whereas transmission success of NC06 was substantially higher in unprimed hosts, for which the NC06 strain was the first MG strain that such hosts had been exposed to (and thus was not truly “heterologous”). Second, if heterology was an important driver of transmission potential during reinfection, transmission of the NC06 strain would have been less affected by our intermediate priming level than was the homologous strain; instead, NC06 appears more strongly affected (relative to unprimed birds) by intermediate priming than was VA94, the homologous strain, although the interaction between strain identity and priming level was not significant. Finally, prior work in this system using larger numbers of MG strains found that strain virulence was a stronger predictor of reinfection likelihood than homology (10, 40), although the effects of homology may result in small increases in acquired protection (10). Overall, our results suggest that inherent strain differences in transmission rate are largely maintained in the presence of strong acquired protection from priming.

Although our two-strain experimental design limits our conclusions to only the two strains used, we can use our results alongside prior work with higher numbers of heterologous strains in this system (10, 40) to speculate as to how detected effects of pathogen priming on transmission potential might ultimately influence virulence evolution in this system. For directly transmitted pathogens, where virulence typically reduces opportunities for transmission (60), high virulence should be favored when it is an unavoidable by-product of the high within-host replication needed for transmission given a contact (20, 21). Thus, any within-host selective environment that requires higher levels of replication to achieve transmission should favor higher pathogen virulence, a prediction supported by prior studies in the mouse-malaria system (22, 23). Because pathogen priming led to significant reductions in pathogen loads and transmission success in reinfected hosts in our study and maximum pathogen loads were associated with successful transmission, a host population with acquired protection from priming should favor a higher optimal pathogen virulence relative to an unprimed, naive host population. Second, optimal virulence is predicted to increase whenever acquired protection in hosts reduces the costs of virulence to pathogens (10, 14) by preventing reinfected hosts from dying when infected with strains of higher virulence. These predictions were confirmed empirically in chickens vaccinated with Marek’s virus (13), which are able to successfully transmit virulent strains in vaccinated flocks. Notably, priming at both intermediate and high levels in our study resulted in significantly lower index bird disease severity during reinfection, and disease severity is a relevant proxy for infection-mediated mortality in this system (61). Thus, while survival did not vary here (because birds survive MG infection in captive conditions where predators are absent), our results suggest that primed hosts in the wild are more likely to survive reinfections, potentially resulting in longer infectious periods for virulent strains in primed hosts (13). Overall, our findings, alongside prior modeling work on this system (10), suggest that acquired protection from priming in this system could favor higher optimal pathogen virulence. However, we emphasize that studies using multiple heterologous strains of varying virulence for reinfections are necessary to test the effects of virulence *per se*, versus other strain characteristics, on transmission potential.

Our results also reveal a key role of pathogen load in driving transmission success among individuals. In contrast to the work by Bonneaud et al. (44), which did not detect the effects of strain-level variation in pathogen loads on MG transmission success, we found that individual variation in pathogen loads was positively associated with pairwise transmission success, regardless of host priming background. Although we also predicted that individual variation in disease severity would correlate with transmission as seen previously in this system (44, 45), we found that associations of disease severity with transmission success depended on a bird’s priming treatment. For intermediate-primed birds, higher disease severity was associated with *reductions* rather than increases in transmission success, such that several birds with both high levels of disease severity and pathogen load did not successfully transmit to cagemates. While the reasons for this are unclear, one possibility is that because birds with high levels of disease severity also show behavioral morbidity during infection (61), high levels of disease severity may sometimes suppress rather than augment transmission opportunities in this system, and likely others (62). Indeed, prior work (45) found that MG-infected house finches that maintained foraging activity were more likely to transmit to cagemates, consistent with the idea that behavioral morbidity can suppress transmission. In contrast, for high-primed birds, disease severity appeared positively associated with transmission success, but due to the strong effects of high priming on protection from disease, we did not have sufficient numbers of high-primed birds with detectable eye scores to estimate this relationship with any precision. Overall, our findings suggest that the effects of disease severity on transmission success differ between reinfected and naive hosts, with potential consequences for virulence evolution.

Overall, our results demonstrate that acquired protection from pathogen priming reduces transmission success during reinfection, potentially in distinct ways for different pathogen strains. These results align with patterns documented in humans reinfected with distinct variants of SARS-CoV-2 (63), a mouse-malaria model (16), and vaccinated chickens challenged with distinct virus strains (13). Together, these patterns emphasize the importance of empirically characterizing how parameters such as host infectiousness vary with acquired protection from vaccination or reinfection and in strain-specific ways. While mathematical models continue to evolve to better capture variation in infection- or vaccine-induced immunity and their consequences for epidemiological dynamics (11, 64–66), such models rarely incorporate reductions in infectiousness for reinfected individuals (see Table 1 in reference 11). Our results and others suggest that such reductions are likely common and represent an important epidemiological variable when characterizing the many systems that fall somewhere between the classically studied susceptible-infected-recovered systems, where recovered individuals acquire complete protection that can wane over time, and susceptible-infected-susceptible systems, where hosts acquire no lasting protection from infection (12). The house finch-MG system offers the opportunity to experimentally test the effects of prior exposure of varying degrees on disease severity and transmission success using an outbred wildlife population and its naturally occurring pathogen, providing an important complement to longitudinal studies of human populations and controlled studies of inbred laboratory or agricultural systems. There are almost certainly key similarities and differences across systems in the role of repeated exposures or vaccinations in contributing to the degree of acquired protection (67, 68) and transmission-blocking immunity (69) in systems characterized by incomplete immunity. Thus, quantifying how epidemiological parameters such as infectiousness vary with acquired protection across a range of host-pathogen systems can improve our ability to understand transmission dynamics, design effective vaccination dosing strategies, and ultimately predict the evolution of more harmful pathogens.

## Data Availability

Data and code used for this study are available in reference 70.

## SUPPLEMENTAL MATERIAL

Supplemental material msphere.00886-24-s0001.docx Fig. S1; additional experimental details.

ASM does not own the copyrights to Supplemental Material that may be linked to, or accessed through, an article. The authors have granted ASM a non-exclusive, world-wide license to publish the Supplemental Material files. Please contact the corresponding author directly for reuse.
