# Supplementary material for: Pathogen priming alters host transmission potential and predictors of transmissibility in a wild songbird species
Source: mSphere. 2025 Mar 10;10(4):e00886-24. doi: 10.1128/msphere.00886-24 (PMC12039224; doi:10.1128/msphere.00886-24)
Supplement: Supplemental material — Fig. S1; additional experimental details. [file msphere.00886-24-s0001.docx]

**Supplemental Materials**


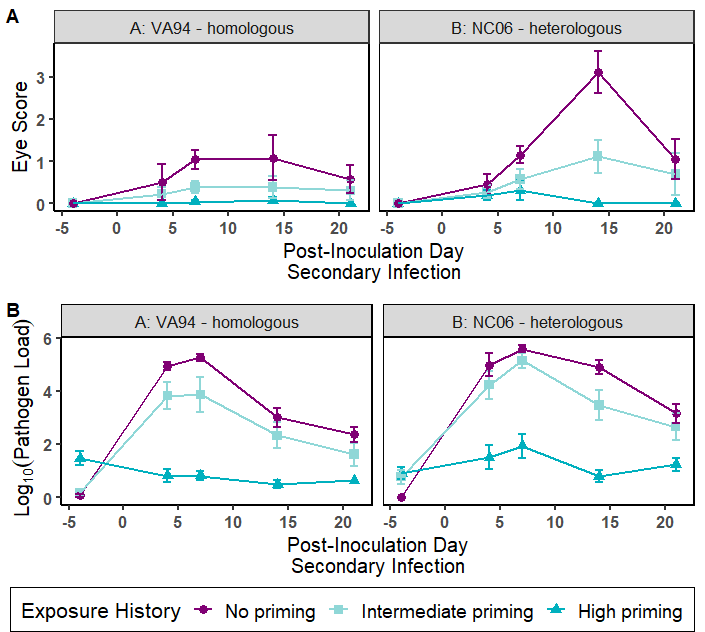


*Figure S1.* A) Disease severity (eye score) and B) pathogen loads over the course of secondary infection for index birds with distinct levels of pathogen priming given a secondary high-dose challenge with one of two strains of *Mycoplasma gallisepticum* (A: VA94; B: NC06). Exposure history (purple, circles – no priming; light blue, squares – intermediate priming; dark blue, triangular points – high priming) largely determined the extent of disease and pathogen load across both strains, although the heterologous strain (NC06) produced higher levels of disease than the homologous strain (VA94), overall. Error bars represent standard error from the mean.

*Bird Capture and Housing*

Hatch-year house finches were captured in Montgomery County, VA in June-July 2016 using a combination of mesh wire traps and mist-nets under permits from VDGIF (056090) and USFWS (MB158404-1). To ensure that birds used in our experiments had no previous exposure to MG in the wild, captive animals underwent a two-week quarantine protocol wherein they were monitored for visible signs of infection and blood sampled on day 14 post-capture to test for MG-specific antibodies (as per (1)). Only individuals that never showed clinical signs of infection, had not been housed with an infected individual, and were seronegative for pathogen-specific antibodies were included in the experiment (n=156 total).

All animals were pair-housed during quarantine, but index birds were single-housed prior to the start of and for the duration of the priming portion of the experiment. After recovery from priming exposures and immediately following secondary challenge, the index birds were then pair-housed with MG-naïve cagemates to assess pairwise transmission potential during reinfection. For the entirety of their time in captivity, finches were held at constant day length (12L:12D) and temperature, and were fed an *ad libitum* diet (Daily Maintenance Diet, Roudybush Inc., Woodland, CA). Individuals were given food in open-cup dishes for the priming portion of the study (when no transmission could occur due to individual housing). When inoculated birds were pair-housed with pathogen-naïve cagemates to quantify pairwise transmission, all pairs were given a two-port hanging tube feeder to mimic the feeder type most likely to facilitate transmission in the wild (2, 3).

*Inoculation*

Stock inocula were grown in Frey’s broth media with 15% swine serum (FMS) and provided by D.H. Ley, North Carolina State University, College of Veterinary Medicine, Raleigh, NC, USA. All inocula were stored at -80°C and thawed and diluted immediately before use. Inoculation dilutions for priming exposures were calculated using the starting viable count of 10^7^ CCU/ml of VA94. To control for the stress of extra handling and inoculation for birds in the repeated low-dose priming group, a randomly selected subset of individuals from the no priming and high-dose priming groups were given a sham inoculation of sterile FMS on priming days 1, 3, 5, 7 and 9 (Fig. 1). No effect of this sham treatment was detected on disease (F = 0.048, df = 30, P = 0.83) or infection outcomes (F = 1.28, df = 12, P = 0.28) compared to control animals not given sham inoculations.

*Pathogen load quantification*

Both conjunctival sacs were swabbed for 5 seconds using separate sterile cotton swabs dipped in tryptose phosphate broth (TPB) and eluted in a single tube containing 300uL of TPB. Samples were kept on ice until frozen at -20°C and remained frozen until thawed for DNA extraction. DNA was extracted using Qiagen DNeasy 96 Blood and Tissue kits (Qiagen, Valencia, CA). Quantitative polymerase chain reaction (qPCR) was performed using a Bio-Rad C1000 CFX96 Real-time System (Hercules, CA). Primers and probes that target the Mgc2 gene of MG were used, and a standard curve of 2.98 x 10^1^ to 2.98 x 10^8^ copy numbers was produced using a plasmid containing a 303 bp Mgc2 insert (4). Cycling parameters used were as follows: 95°C for 3 minutes then 40 cycles of 95°C for 3 seconds followed by 60°C for 30 seconds (5).

1. Hawley DM, Grodio J, Frasca S, Kirkpatrick L, Ley DH. 2011. Experimental infection of domestic canaries (Serinus canaria domestica) with Mycoplasma gallisepticum: a new model system for a wildlife disease. Avian Pathol 40:321–327.

2. Adelman JS, Moyers SC, Farine DR, Hawley DM. 2015. Feeder use predicts both acquisition and transmission of a contagious pathogen in a North American songbird. Proceedings of the Royal Society B: Biological Sciences 282:20151429.

3. Hartup BK, Mohammed HO, Kollias GV, Dhondt AA. 1998. Risk factors associated with mycoplasmal conjunctivitis in house finches. J Wildl Dis 34:281–288.

4. Grodio JL, Dhondt KV, O’Connell PH, Schat KA. 2008. Detection and quantification of Mycoplasma gallisepticum genome load in conjunctival samples of experimentally infected house finches (Carpodacus mexicanus) using real-time polymerase chain reaction. Avian Pathol 37:385–391.

5. Hawley DM, Osnas EE, Dobson AP, Hochachka WM, Ley DH, Dhondt AA. 2013. Parallel patterns of increased virulence in a recently emerged wildlife pathogen. PLoS Biol 11:e1001570.
